# Functional Connectivity Between Human Motor and Somatosensory Areas During a Multifinger Tapping Task: A Proof-of-Concept Study

**DOI:** 10.3390/neurosci7010012

**Published:** 2026-01-14

**Authors:** Roberto García-Leal, Julio Prieto-Montalvo, Juan Guzman de Villoria, Massimiliano Zanin, Estrella Rausell

**Affiliations:** 1 Ph.D. Program in Neuroscience, Universidad Autónoma de Madrid-Cajal Institute, 28029 Madrid, Spain; rgarcia_leal@yahoo.es; 2 Department of Neurosurgery, Hospital General Universitario Gregorio Marañón, 28007 Madrid, Spain; 3 Instituto de Investigación Sanitaria Gregorio Marañón, 28007 Madrid, Spain; 4 Department of Neurophysiology, Hospital General Universitario Gregorio Marañón, 28007 Madrid, Spain; 5 Facultad de Medicina, Universidad Complutense de Madrid, 28040 Madrid, Spain; 6 Department of Radiology, Hospital General Universitario Gregorio Marañón, 28007 Madrid, Spain; 7 Instituto de Física Interdisciplinar y Sistemas Complejos CSIC-UIB, 07120 Palma de Mallorca, Spain; 8 Department of Anatomy, Histology and Neuroscience, School of Medicine, Autónoma University of Madrid, 28029 Madrid, Spain

**Keywords:** motor cortex, somatosensory cortex, Area 3a, information imbalance gain causality, fMRI, tapping task

## Abstract

Hand representation maps of the primate primary motor (M1) and somatosensory (SI) cortices exhibit plasticity, with their spatial extent modifiable through training. While activation and map enlargement during tapping tasks are well documented, the directionality of information flow between these regions remains unclear. We applied Information Imbalance Gain Causality (IIG) to examine the propagation and temporal dynamic of BOLD activity among Area 4 (precentral gyrus), Area 3a (fundus of the central sulcus), and SI areas (postcentral gyrus). Data were collected from both hemispheres of nine participants performing alternating right–left hand finger tapping inside a 1.5T fMRI scan. The results revealed strong information flow from both the precentral and postcentral gyri toward the sulcus during tapping task, with weaker bidirectional exchange between the gyri. When not engaged in tapping, both gyri communicated with each other and the sulcus. During active tapping, flow bypassed the sulcus, favoring a more direct postcentral to precentral way. Overtime, postcentral to sulcus influence strengthened during non task periods, but diminished during tapping. These findings suggest that M1, Area 3a, and SI areas form a dynamic network that supports rapid learning processing, where Area 3a of the sulcus may contribute to maintaining representational plasticity during complex tapping tasks.

## 1. Introduction

It is now well established that, in the central nervous system, representational maps of the motor and primary somatosensory cortices exhibit substantial plasticity, with their spatial extent modifiable through training. Although the mechanisms underlying such dynamic change remain incompletely understood, those are very likely influenced by several factors related to the somatic experience accompanying repeated movements. These include the modality of the sensory input received, the degree of convergence of different inputs onto individual neurons, the internal cortico-cortical circuits that interconnect movement-coordinated neurons across cytoarchitectonic areas, and training-induced modifications of synaptic transmission within those networks [1]. Collectively, these processes result in a more efficient recruitment of the motor network. As will be briefly reviewed below, extensive knowledge on those mechanism has been gathered from electrophysiological and tract tracing studies in monkeys, and more recently from neuroimaging studies in humans—albeit the latter ones with the inherent limitation of imaging resolution. Yet, even if physiological properties of that plasticity and their structural connectivity in both species were fully characterized, the temporal dynamic events and functional connectivity that unfold during information flow between the motor and somatosensory areas throughout training have not been systematically investigated. This study aims to systematically characterize the functional connectivity linking the primary motor and somatosensory cortices during a two-hand training paradigm in which participants sequentially tap their fingers against the thumb, alternatively engaging the right and left hemispheres while undergoing fMRI scanning.

Each of the four primary somatosensory cytoarchitectonic areas—Areas 3a, 3b, 1 and 2—[2,3,4,5,6] contains an independent, systematic representation of peripheral receptors, with the lower extremities represented dorsomedially and upper extremities ventrolaterally. In monkeys, the somatotopic maps of Areas 3b and 1 are the most precise and detailed [7,8,9,10,11]. Lesion and electrode tract-tracing studies in monkeys [12,13] and high-resolution Cerebral Blood Volume fMRI [14] have shown that the modal specificity and receptive field properties of Areas 3a, 3b, 1, and 2 are supported through two parallel pathways. The first involves thalamo-cortical connections targeting mainly granular layers: Area 3a receives inputs primarily from muscle stretch receptors, Area 3b from rapidly and slowly adapting cutaneous receptors, Area 1 from rapidly adapting cutaneous receptors, and Area 2 from deep pressure receptors. The second pathway consists in convergent cortico-cortical projections amongst pyramidal layers, whereby neurons from Area 3a and 3b project to Areas 1 and 2, enabling the latter two to integrate information from different receptors. Across successive stages of sensory processing, neuronal response properties become progressively more complex and the receptive fields increase in size, reflecting integration of inputs. Neurons in Area 3a respond mainly to joint manipulation or other types of deep stimuli, whereas those in Area 3b respond mainly to simple punctual cutaneous stimuli. In contrast, neurons in Areas 1 and 2 can be motion-, direction-, or orientation-sensitive. Receptive fields in Areas 3a and 3b are small -typically limited to one or two finger phalanges-, while those in Areas 1 and 2 are considerably larger, often encompassing one or several adjacent fingers [15,16,17,18,19,20].

The early descriptions of the somatotopy of the human primary somatosensory cortex (SI) depicts the tactile representation of the body surface along the postcentral gyrus, with the digits arranged from D5 (little finger) to D1 (thumb) in medial-to-lateral sequence [21,22]. Numerous subsequent studies have confirmed this arrangement using functional magnetic resonance imaging (fMRI) during tactile or electrical stimulation of the distal phalanxes [23,24,25,26]. High-resolution 3T fMRI studies have further resolved the representations of all phalanges and digit bases of all fingers in SI [27]. Using 7T fMRI and a travelling wave paradigm, Ref. [28] has mapped the internal somatotopic representation of the index, middle, and ring fingers in human S1 with high spatial resolution and robust BOLD contrast. Their results revealed multiple map reversals at the tip and base that correspond to the borders between Brodmann Areas 3a, 3b, 1, and 2. Maps exhibit consistent plasticity, with their spatial extent modifiable through training, that has been well documented. A recent 7T fMRI study in humans by Spencer et al. [29] demonstrated that digit representations of Area 3b remain quite stable and that training-induced changes primarily involve interactions between pairs of neighbouring fingers. In contrast, Areas 4 and 2 exhibit stronger and more wide spread interactions, including those between non-adjacent fingers and coordinated changes involving triplets and quadruplets. Area 4, the primary motor cortex (M1), has traditionally been viewed as an executive locus for simple voluntary movements, sending commands to individual muscles [30]. However, data from animal studies and human neuroimaging studies suggest that M1 generates more complex commands related to the conception and organization of actions rather than individual muscle activation [31,32,33,34,35]. Based on PET findings, Ref. [36] further proposed that human M1 contributes to preparation of movements, particularly for reaching, and to motor learning. Studies using fMRI [37] and single-pulse trans-cranial magnetic stimulation [38] have shown that motor learning of movement sequences induces changes in M1 representations of hand muscles. A recent fMRI study demonstrated that the planning of finger movements activates regions in Area 4 and SI, with peak activity patterns that closely match those elicited during actual movement execution [39]. 7T Spin-echo BOLD fMRI has defined spatial specificity in the human motor cortex during finger movement tasks [40] very well. More recently, spontaneous activity in human motor cortex has been shown to form fine-scale, patterned representations associated with behaviors frequently performed in daily life [41]. Everyday motor planning and behavior then depend on the continuous interplay between complex motor control and precisely timed somatosensory feedback. Although many anatomical studies have demonstrated dense cortico-cortical connections between M1 and SI, the functional mechanisms by which somatosensory signals functionally interact with the motor Area to guide natural hand movements remain largely unknown.

Learning and motor plasticity are thought to depend in part on intracortical interactions between motor and somatosensory maps. Direct and reciprocal connections have been well documented in rodents [42,43,44,45,46], cats [47,48,49,50], and macaques [13]. Using anterograde and retrograde tracers, Ref. [13] mapped projections between Areas 1, 2, 3 (S1), 4 (M1), and 5 in the forelimb representation of monkeys. They reported that Area 3b is not connected to Areas 3a or 4, but projects to Areas 1 and 2; that Area 1 is reciprocally connected with Areas 3a and 3b; and that Area 2 is reciprocally connected with areas 4 and 3a. Although few additional track-tracing studies have been conducted since then in monkeys, recent functional works has begun to explore the dynamic interactions between SI and M1 during hand movements. Using high-density multi-electrode arrays, Ref. [51] recorded single unit activity and local field potentials from rostral and caudal portions of M1 and Areas 3a and 2 during grasping. Their findings showed that M1 and SI sites with similar receptive or projection field were more likely to be functionally coupled, suggesting that such connections support and facilitate the synergistic coordination of movement with sensation. Large-scale analyses with Human Connectome Project data have further characterized this circuits [52,53,54]. By using diffusion tractography, these studies quantified structural connections across cortical regions; functional connectivity was assessed through correlations in resting state BOLD signals; and effective connectivity was estimated using the Hopf model to infer the strength and direction of the causal connectivity of causal interactions. These analyses revealed strong effective and functional connectivity between M1 and SI, particularly between Areas 3a and 3b towards Areas 1 and 2. More recently, a study investigating motor learning examined whole brain functional connectivity with motor cortex during implicit and explicit manual task learning, finding strong interactions between M1 and SI [55]. Despite all these advances, functional connectivity within SI and between M1 and SI during execution of single motor task, like tapping during fMRI, has not yet systematically been assessed.

Functional connectivity between fMRI voxel-time series is usually inferred by quantifying the associations between every pair of time series. Bivariate methods offer fine-grain voxel-level resolution, but often also introduce spurious indirect connections, whereas while multivariate methods operate at a coarse regional scale and therefore miss voxel-level details [56]. Techniques such as Granger Causality (GC) and Large-Scale Granger Causality overcome these limitations estimating direct information flow at the voxel level in a multivariate network, avoiding undetermined and overly complex model spaces. These analytical tools have been successfully used to characterize dynamic networks in which functionally related fields interact [56], and to determine directional influences in reciprocal cortico-cortical connections, for example, between frontal and parietal regions during resting state [57]. Granger Causality (GC) has been extensively applied to investigate motor networks. For instance, it has been found that during self-paced finger tapping, activity changes in the premotor cortex are predicted by earlier fluctuations in M1 and are modulated by the supplementary motor area and the anterior precuneus [58]. Other studies have reported strong GC links between voxels of the left motor cortex and the supplementary motor area [59], and between M1 and the cerebellum during right-hand tapping [60]. EEG studies found distinct cortical network for Gamma Synchronization in voluntary hand movement tasks suggesting that SIM1 modulated the activity of interconnected cortical areas through Gamma Synchronization, while subcortical structures modulated the motor network dynamically, and specifically for the studied hand movement [61]. Multimodal studies combining fMRI, fNIRS, and EEG further show bi-directional effective connectivity within a broad cortico-cortical sensorimotor network involving several areas (premotor cortex, supplementary motor area, and dorsolateral prefrontal cortex) during finger movement tasks, with consistent GC findings across modalities [62]. GC predictions of activity have also been used to compare the functional connectivity during motor imagery in stroke patients, providing insight into disrupted motor networks and their potential for rehabilitation [63,64].

**Figure 1:**
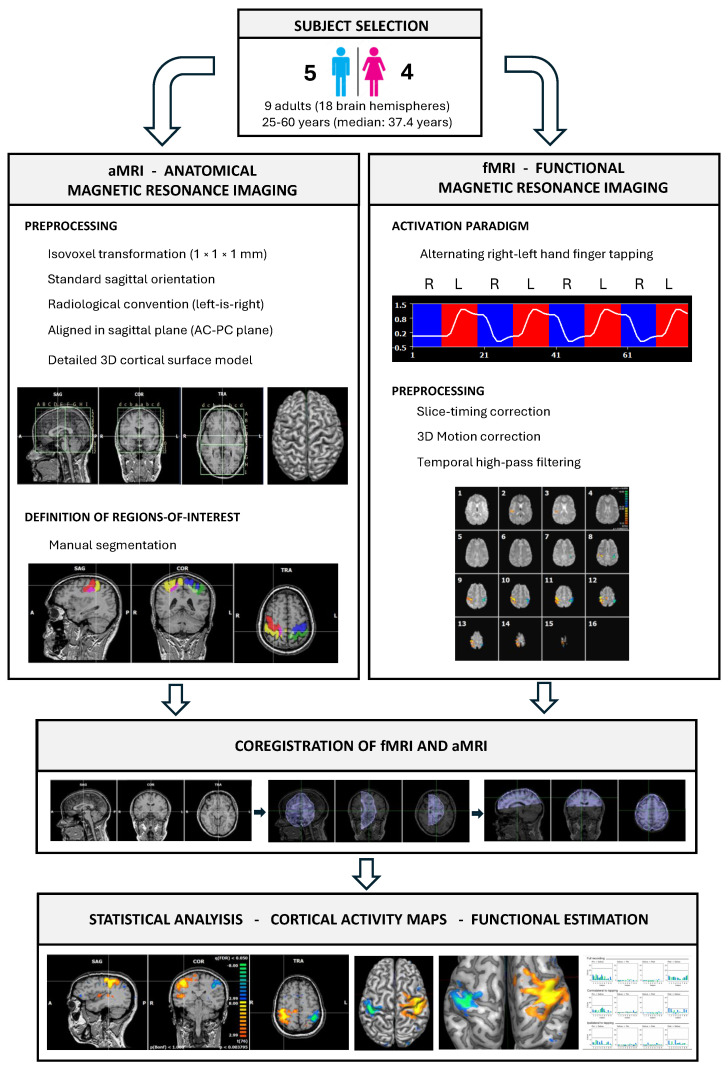
Overview of the main steps of data recording and processing. See Section 2.1 for details.

Our aim here was to investigate the fMRI functional connectivity between the primary motor cortex (Area 4 in the precentral gyrus and anterior lip of the central sulcus), the region at the fundus of the central sulcus (Area 3a of SI), and the areas included in the posterior lip of the central sulcus and in the postcentral gyrus (Areas 3b, 1, and 2 of SI) during a complex finger sequence tapping task in humans [65]. In order to retain the micro-scale information at the level of individual voxels, we here employ a recently proposed causality metric, called Information Gain Imbalance [66]; the results are further compared with classical causality measures, including Granger Causality [67] and time-delayed Mutual Information. These techniques have been applied to a data set of fMRI recordings obtained from healthy volunteers, recorded in a clinical setting and while performing the aforementioned task (see Section 2.1 and Figure 1 for details). The results, reported in Section 3, indicate a strong information flow from the precentral and postcentral gyri to the sulcus, albeit with an intensity evolving in time. Specifically, the postcentral gyrus increasingly transfers information to Area 3a of the fundus of the central sulcus over time, suggesting a role for this region during periods of non task engagement. However, during active tapping, information flow bypasses the sulcus in favor of a more direct and faster postcentral to precentral pathway. As will be discussed below, these findings support the role of M1, Area 3a, and SI areas in the dynamic network involved in fast learning processing, while Area 3a of the sulcus may contribute to maintaining representational plasticity during complex sequential tapping tasks.

## 2. Materials and Methods

### 2.1. Subject Recruitment and Data Recording

#### 2.1.1. Subjects Selection

Anatomical and functional magnetic resonance imaging (MRI) studies were performed in 9 right-handed healthy volunteers (four women and five men), 25 to 60 years old (mean 37.4 years)—see Table A1. Informed consents were obtained from all subject after the nature of the procedure had been fully explained. The study was conducted in accordance with institutional guidelines and received approval from de Ethics Committee for Clinical Research of the Instituto de Investigación Sanitaria Gregorio Marañón, former Fundación para la Investigación Biomédica del Hospital Gregorio Marañón (protocol code 15/2006 613 on 13 December 2006).

#### 2.1.2. Imaging Procedure

For each participant, anatomical and functional brain MRI examinations were performed on a 1.5 T scanner (Philips Medical Systems Intera^®^, Philips Medical Systems, Best, The Netherlands) using the following parameters:

- Anatomical MRI (aMRI): Three-dimensional gradient-echo volumetric T1-weighted sequence (flip angle 30°; echo time (TE) of 4.6ms; matrix size 256×256; slice thickness of 1.5 mm.
- Functional MRI (fMRI): BOLD-sensitive multi-slice echo-planar imaging (EPI) sequence (EPI factor 63, single-shot) with gradient-echo preparation (flip angle 90°, repetition time (TR) =3000 ms, echo time (TE) =50 ms); matrix size 64×64, field of view (FOV) of 230 mm.

The head was fixed within the standard head coil to minimise head motion during the study. Subjects were instructed to relax and keep their eyes closed during the scanning procedure. They were also instructed to move only their fingers during execution of the motor task, with their forearms resting comfortably on their thighs.

#### 2.1.3. Activation Paradigm

The motor task consisted of a finger tapping protocol based on [65], in which all fingers are sequentially tapped, from finger to the thumb, in a repetitive manner, as described in several previous studies [68,69,70,71,72]. Each stimulation run consisted of four acquisitions (30 s per acquisition) of the activation condition (right hand finger tapping) and four acquisitions (30 s per acquisition) of the control condition (left hand finger tapping) [65]. This resulted in a total acquisition time of 4 min per run. The movement was self-paced, following the instruction to move the fingers as fast as comfortably possible without exerting extra force. The beginning and end of each period were announced via the microphone using the commands left, right, and stop. This protocol is easy to perform inside the scanner and is widely used in neuroimaging studies of the human motor system. Its effectiveness has been validated in numerous studies [65,68,69,70,71,72,73], and reliably activates a large extent of the medio-lateral domain of sensorimotor regions. Alternating the task between the right and left hands allowed us to distinguish the involvement of each hemisphere during active tapping of the contralateral hand, from when tapping is performed by the ipsilateral hand.

#### 2.1.4. Image Preprocessing

All anatomical and functional images (aMRI and fmRI) obtained were processed using the BrainVoyager QX v2.8 software (Brain Innovation, Maastricht, The Netherlands), under the corresponding license. The DICOM data from the aMRI images were processed to perform an iso-voxel transformation (1×1×1 mm matrix) and were converted to a standard sagittal orientation. Radiological convention (left-is-right) was used. All aMRI data sets were aligned in the sagittal plane using a plane passing through the anterior and posterior commissures (AC-PC plane). A detailed three-dimensional reconstruction of the cerebral cortex was constructed from the aMRI through a series of preprocessing and surface reconstruction steps, including correction for intensity inhomogeneities; segmentation to separate white matter, gray matter, and cerebrospinal fluid; and identification of the gray–white matter interface and generation of a surface along the pial boundary to obtain a detailed 3D cortical surface model suitable for visualization of cortical activity maps.

#### 2.1.5. Definition of Regions of Interest

Regions of interest (ROIs) were manually defined in BrainVoyager QX v2.8 using the anatomical segmentation tools provided by the software. We have investigated three regions of BOLD fMRI activation in the primary motor and somatosensory cortices. The precentral gyrus (Brodmann area 4), the fundus of the central sulcus (Area 3a), and the postcentral gyrus (Areas 3b, 1 and 2) were identified on the aMRI dataset, by examining sagittal, coronal, and axial views and tracing their boundaries slice by slice following Geyer et al.’s definition [74,75]. These authors reported that Brodmann Area 4 is located in the precentral gyrus and the anterior wall of the central sulcus; Area 3a lies in the fundus of the central sulcus; Area 3b is located in the rostral bank of the postcentral gyrus (posterior wall of the central sulcus); Area 1 lies on its crown; and Area 2 reaches down into the postcentral sulcus and its rostral wall. Manual segmentation was performed voxel by voxel. We manually outlined the borders, as interareal borders do not align with macrostructural landmarks and exhibit considerable inter-individual variability [74]. The resulting ROIs were saved as volume-of-interest (VOI) files and subsequently used for functional data extraction for statistical analysis.

#### 2.1.6. Fmri Preprocessing

The DICOM data from the fMRI images were pre-processed in order to reduce noise and prepare the data for statistical analysis using a sequence of standard correction procedures available in the BrainVoyager QX v2.8 software [76,77]:

1. Slice-timing correction to compensate for temporal differences in slice acquisition [78].
2. Three-dimensional motion correction to adjust for head movements across volumes [79,80].
3. Temporal high-pass filtering to remove slow signal drifts [81,82,83].

After these steps, the preprocessed time series are ready for a general linear model (GLM) specification using the activation paradigm (protocol), and further statistical analysis.

#### 2.1.7. Coregistration of Functional (fMRI) and Anatomical (aMRI) Data Sets

After the initial preprocessing of both data sets, as described in the previous paragraphs, the functional data set (fMRI) was coregistered to the anatomical volume (aMRI) using intensity-based rigid-body alignment following standard procedures [76,77,79]. The functional data set is resampled into the anatomical space, ensuring precise spatial correspondence between anatomical structures and functional activation maps. This coregistered data set was subsequently used for statistical modeling.

#### 2.1.8. Statistical Analysis and Generation of Activation Statistical Maps

**Figure A1:**
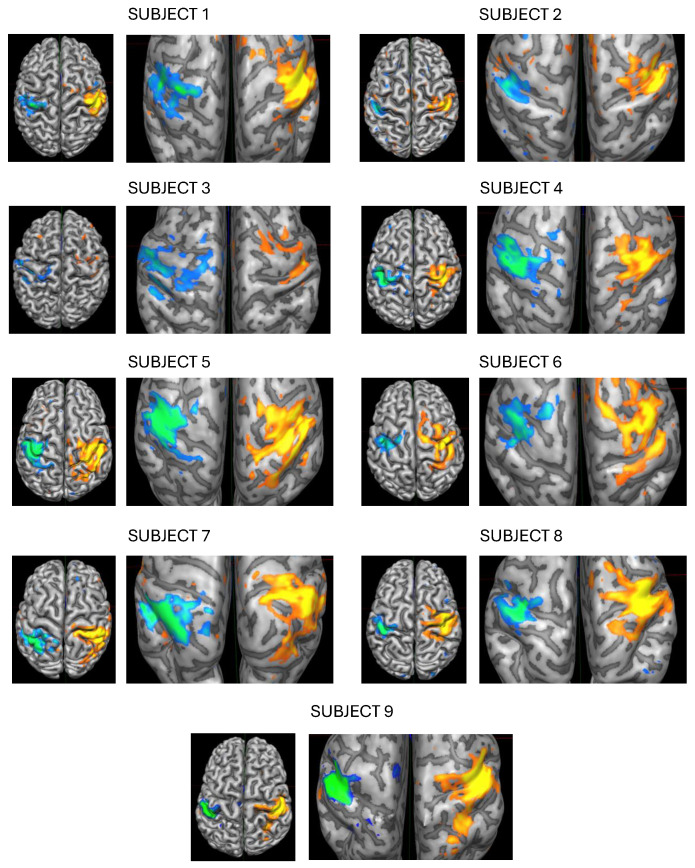
Graphical representations of the statistical activation maps of all subjects during the tapping—see Section 2.1.8 for details. For each subject, these maps are reported embedded over (**left**) a detailed 3D model of the brain surface, as obtained by the aMRI, and (**right**), the insuflate surface, with a zoom on the three regions of interest. Colors, from blue to green (left hemisphere) and from red to yellow (right hemisphere), represent the degree of significance.

For each subject, the preprocessed fMRI tine series were analysed using a voxel-wise general linear model (GLM) approach to quantify task-related BOLD signal changes across the brain, following the methodology originally described in Refs. [84,85], and detailed in BrainVoyager QX v2.8 user’s guide [76,77]. Critically, we acknowledge known limitations of the GLM approach [86,87]. Experimental conditions were modeled using a block design, with each condition represented by a boxcar predictor defined in protocol files and convolved with a canonical hemodynamic response function. The GLM was estimated voxel-wise on the volume time-course data to obtain b-weights for each predictor, representing the contribution of each predictor to the observed time course. Statistical parametric maps were generated by computing t-contrasts of interest. The resulting t-maps, which identified brain regions significantly engaged during the task, were overlaid on the individual anatomical volumes (aMRI) and on 3D cortical surface models, and thresholded at the voxel level—see Figure A1 for a graphical representation.

Following the statistical analysis, quantitative data were extracted from three anatomically defined volumes of interest (VOIs): the precentral gyrus, the fundus of the central sulcus, and the postcentral gyrus. For each subject, Volume–Time Course data were obtained by sampling the fMRI signal within these VOIs across the entire experiment, yielding region-specific time series aligned with the experimental design. In addition, Object Details tables were generated from the statistical maps, providing numerical information for each cluster within de VOIs (cluster size, peak voxel coordinates, and peak t-values). These tabular data were exported for subsequent statistical analysis.

### 2.2. Functional Estimation

In this work we leverage a recently proposed metric of causality, called Information Imbalance Gain (IIG), whose results are further compared with standard approaches for the reconstruction of functional relations—specifically, Granger Causality and Mutual Information. The three of them are based on the concept of “predictive causality”: a region *X* is assumed to be causing another region *Y* if knowledge of the former helps predicting the future dynamics of the latter. At the same time, the specific way of evaluating this causality strongly varies. For the sake of completeness, these are briefly described here below.

- Information Imbalance Gain (IIG) Causality. This causality test builds on top of the Information Imbalance, a recently proposed statistical measure aimed at comparing whether the information contained in two distance sets $d_{X}$ and $d_{Y}$ are equivalent, or on the contrary, if one is more informative than the other [88]. The IIG test then relies on the idea that, if *X* is causing *Y* and one is trying to predict the future state of *Y*, a distance measure built using the present states of both *X* and *Y* will have more informative than a distance built using only *Y*. It derives that, in the presence of a causality, the Information Imbalance between the past and future of *Y* alone should be larger than that of a combination of the past of *X* and *Y*, and the future of *Y*. This is then assessed by calculating Euclidean distances, and the ranking of between them. A full definition of this IIG test is reported in [66]. As a final step, the observed imbalance is compared against what obtained in a large number (here, 100) of random permutations of *X*, i.e., when all causal relationships are artificially deleted. The magnitude of such deviation is expressed as a Z-Score. Note that, by construction, this Z-Score is the result of a random process, and thus is more correctly represented by a distribution—see Appendix B for a discussion.
- Mutual Information (MI). The MI I(Y;X) between two random variables *X* and *Y* is defined as the expected reduction in uncertainty about *Y* resulting from knowledge of the value of *X*. Mathematically this is given by
  (1) $$I\left(Y;X\right)=-\sum_{y}\sum_{x}p\left(x,y\right)\log\left(\frac{p(x,y)}{p(x)p(y)}\right),$$
  with p(x,y) being the joint probability distribution, and p(x) and p(y) the marginal probability distributions. The MI is here calculated considering a lag of one time point; in other words, we quantify how much the uncertainty about the dynamics of one region y(t) at time *t* is reduced by knowing the past dynamics of another region x(t−1) at time t−1. Thus, while the MI is not a causality metric per se, the use of a time lag allows to estimate such kind of time-asymmetric relationships. Additionally, the MI has been calculated using a box kernel density estimation procedure [89], using a bandwidth of 0.2, and following the implementation included in the *infomeasure* Python package (version 0.5.0) [90]. Finally, a *p*-value is obtained through a permutation hypothesis testing, in which the *X* time series is randomly permuted to destroy any temporal information.
- Granger Causality (GC). The GC test is based on testing the hypothesis that, whenever an element *X* is causing *Y*, including information about the dynamics of the former helps to predict the future of the latter—or, in other words, that *X* contributed in defining the future of *Y*. Contrary to the MI, the GC tests this hypothesis by constructing two autoregressive-moving-average (ARMA) models, forecasting the future dynamics of *Y* by, respectively, including or not information about the past of *X* [67]. Finally, an F-test is performed on the residuals, i.e., on the errors of the predictions, to confirm whether introducing additional information results in a reduction of these.

It is important to highlight two important differences between these three causality tests: Firstly, as previously mentioned, the IIG test yields a Z-Score, while the GC and MI directly return a *p*-value. This will affect how results obtained for multiple subjects will be aggregated, as will be discussed in Section 2.3.

Secondly, both the GC and MI analyse univariate time series; in other words, the activity recorded for the set of voxels belonging to a region has to be averaged out to obtain a single time series of its global dynamics. The detection of the causality between two regions is then performed by analysing the corresponding time series, as discussed above. Note that, while multivariate versions of both tests exist, the large number of voxels in each region makes this approach computationally unfeasible. On the other hand, the IIG test considers all individual voxels in a given region, and compares the distance between them. To illustrate, the distance $d_{X,Y}$ between the activity of voxels of *X* and *Y* is given by the Euclidean distance: $\left(|\right|X^{i}-X^{j}{\left|\right|}^{2}+\left|\right|Y^{i}-Y^{j}{\left|\right|}^{2}{)}^{1/2}$. Here, *i* and *j* represent different instances, for instance the activity recorded in different moments in time—see Ref. [66] for a complete discussion. In synthesis, while GC and MI analyse the average activity of regions, the IIG test accounts for the activity of all voxels to them belonging; consequently, the latter one is expected to be more sensitive, especially when short recordings are available.

### 2.3. Statistical Analysis

When evaluating the aforementioned functional metrics, the results are a set of values indicating the statistical significance, i.e., either a Z-Score (for IIG) or a *p*-value (GC and MI). As multiple independent values are obtained in each case, i.e., one for each subject and tapping side, we further aggregate them into a single value representing the significance of the information flow across all the experiments.

We firstly consider Stouffer’s Z-Score method, according to which the Z-Score of the full (or meta-) analysis can be obtained as

(2) $$Z∼\frac{\sum_{i=1}^{k}Z_{i}}{\sqrt{k}},$$

where $Z_{i}$ is the Z-Score of the *i*-th individual analysis, and *k* is the number of individual analyses. The aggregated Z-Score is, thus, defined as the average value normalised using the square root of available instances.

Secondly, we use Fisher’s method to combine multiple *p*-values into one test statistic as

(3) $$X_{2k}^{2}=-2\sum_{i=1}^{k}\ln p_{i},$$

where $p_{i}$ is the *p*-value for the *i*-th hypothesis test. When all the tests are independent, the above statistic has a chi-squared distribution with 2k degrees of freedom, with *k* being the number of tests being combined. From this, the final aggregated *p*-value is finally obtained.

## 3. Results

### 3.1. IIG Causality

**Figure A3:**
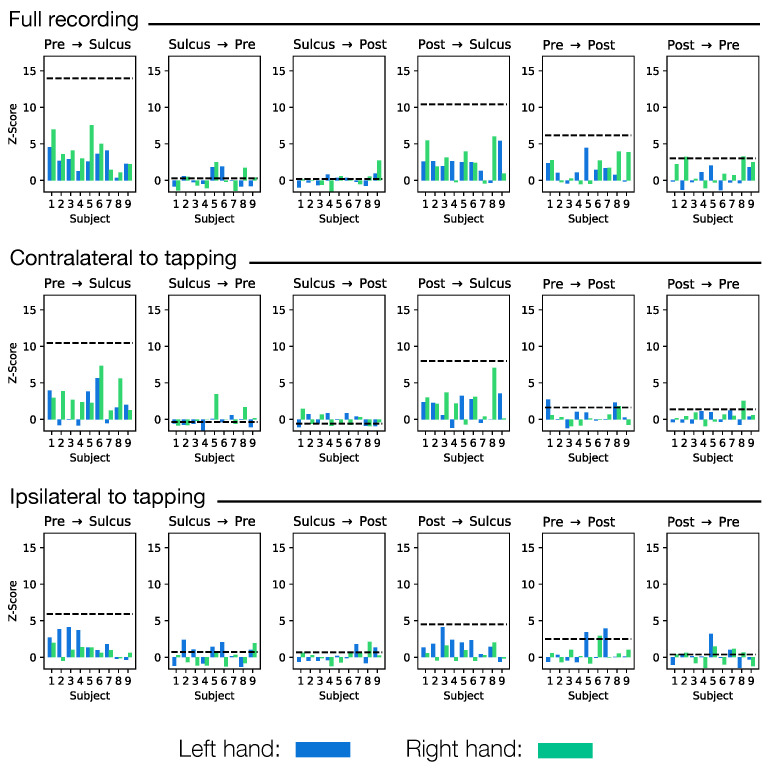
Individual and aggregated Z-Scores obtained using the IIG causality test. Each panel reports the results for a pair of regions (see titles). From top to bottom, each set of panels, respectively, corresponds to the whole recording; to the activity of the hemisphere contralateral to the tapping; and to the activity ipsilateral to the tapping. Inside each panel, the blue and green bars indicate the Z-Score obtained for each participant, respectively, for the left and right hand. Finally, the horizontal dashed lines report the aggregated Z-Score, as described in Section 2.3.

We start the analysis of the functional connectivity by using the IIG causality, as previously described in Section 2.2. For the sake of conciseness, the main results, i.e., the aggregated Z-Score between each pair of regions, are reported in Table 1; on the other hand, full details, including the Z-Score for each subject and side, are included in Appendices, see Figure A3 and following.

**Figure A4:**
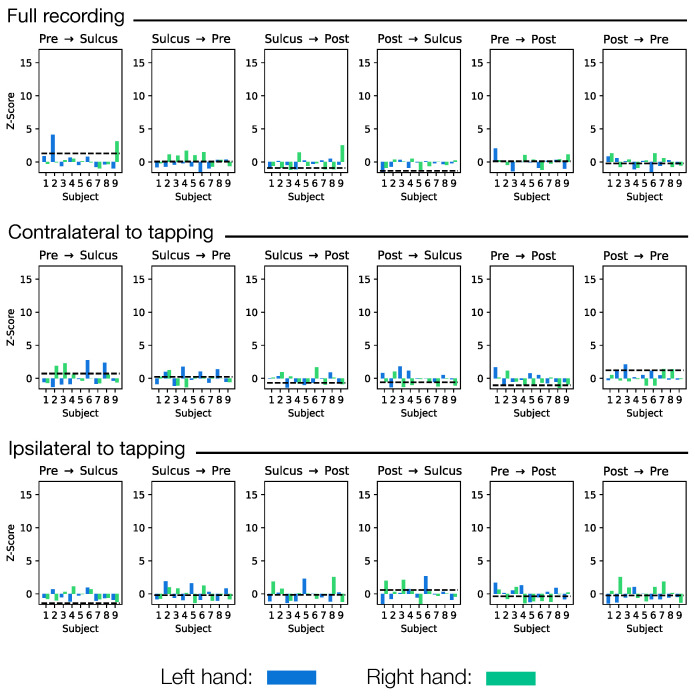
Individual and aggregated Z-Scores obtained using the IIG causality test on the randomly shuffled data. Meaning of panels, bars, and horizontal lines as in Figure A3.

When considering the full recording, i.e., when not differentiating between tapping and resting, two main flows of information are detected by the IIG causality, respectively, starting in the precentral and postcentral gyri, and both ending in the sulcus. For these two cases this test yields aggregated Z-Scores of, respectively, 13.97 and 10.41, see the top part of Table 1. Notably, these are completely lost when the corresponding time series are randomly shuffled, thus excluding the possibility of being false positives—see Figure A4. This is also not a consequence of the different number of voxels present in each region, see Appendix B for a discussion. In addition, weaker but still statistically significant propagations can be observed from the precentral to the postcentral gyri (Z-Score of 6.17), and vice versa (Z-Score of 3.02).

We further apply the functional metric to the parts of the time series that describe the activity of the hemisphere contralateral to the tapping, and to those ipsilateral to it. The former ones, thus, correspond to the response to the tapping activity alone, while the latter ones to time periods in which that hemisphere is not directly involved in the task. As can be seen in the middle and bottom parts of Table 1, the two main relations (precentral → sulcus and postcentral → sulcus) are maintained in the contralateral side, but are weakened in the ipsilateral case. Additionally, the precentral → postcentral flow is partly maintained in the ipsilateral case, while the postcentral → precentral one is mostly lost, remaining in the contralateral case in a non-significant way.

**Figure 2:**
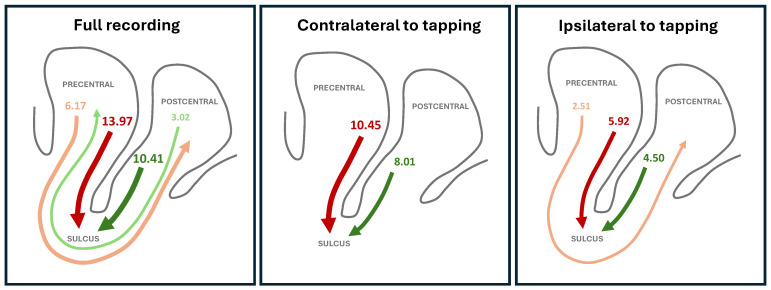
Graphical representations of the main flows of information detected by the IIG causality—see also Table 1. Line thickness is proportional to the aggregated Z-Score, also reported numerically.

These results, also graphically illustrated in Figure 2, seems to point towards a strong connection from the pre- and postcentral gyri to the sulcus, and also a weaker connection between both gyri. Due to the low temporal resolution of fMRI data, a complete picture of the flow of information cannot be recovered. Specifically, two scenarios may be behind these results. In the first one, the sulcus receives information from the two gyri, and then retransmits it back to them. If the second transmission is done with a speed comparable to the temporal resolution of the time series, what is observed will mainly be a direct flow between pre- and postcentral gyri, and vice versa. Note that the IIG test would exclude information flows that happen faster than the time resolution of the time series, as these would appear as instantaneous correlations and not true causalities. In the second scenario, the pre- and postcentral gyri are directly exchanging information between them, in addition to transmitting to the sulcus. The coexistence of these two results is further exemplified in Appendix D using a synthetic model of fMRI dynamics.

**Figure 3:**
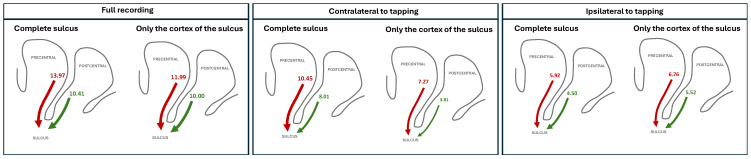
Graphical representations of the main flows of information detected by the IIG causality, comparing when the activity of the white matter of the sulcus is included (left graph) or excluded (right graph). See Table 2 for full numerical values. Line thickness is proportional to the aggregated Z-Score, also reported numerically.

Notably, our initial definition of the sulcus also includes voxels of white matter, through which fibers connecting the two gyri are passing; it is, thus, possible that the causality detected between the gyri and the sulcus is the result of including signals passing through these fibers. In order to assess this possibility, Table 2 reports the aggregated Z-Scores obtained by the IIG test when only voxels belonging to the cortex are considered as part of the sulcus; see also Figure 3 for a graphical representation. It can be appreciated that results are generally similar, albeit with a reduction of the Z-Scores for precentral → sulcus and postcentral → sulcus in the contralateral case.

In synthesis, these results seem to point towards a complex connectivity scenario. During time periods in which the hemisphere is not directly involved in the tapping, pre- and postcentral gyri exchange information both between themselves and with the sulcus (see values in Table 1). This exchange of information seems to be mediated by the sulcus, as can be seen by the fact that excluding the white matter of the latter has little impact (see last column of Table 2); in other words, the direct connection between the two gyri, if present, is not affecting the causality test. On the other hand, while being involved in the tapping, the flow of information between the two gyri seems more direct. This is highlighted by the fact that removing the while matter from the definition of the sulcus has a significant impact (see central column of Table 2). Additionally, the IIG test yields much lower Z-Scores, possibly indicating that this flow, being direct and not mediated, is faster than the time resolution of the time series. Still, these results have to be evaluated in the light of the inherent limitations of the data and the tapping task, as will be discussed below.

### 3.2. Alternative Functional Metrics

As previously introduced, while the main results were obtained using the IIG causality, the same analyses have been repeated using the MI and GC metrics, as examples of alternative approaches common in the literature. While being conceptually simpler, the present two important drawbacks: Firstly, as already introduced in Section 2.2, they are calculated considering the average time series of the activity of each region, and not the activity of individual voxels. While the latter option could in principle be possible, the high number of voxels, and especially the high ratio between voxels and time steps in the series, precludes its viability. Secondly, these two tests analyse the whole time series, as opposed to sequential values in them; again due to the limited number of time points available in the series, statistically significant results can only be obtained for the whole time series.

**Figure A7:**
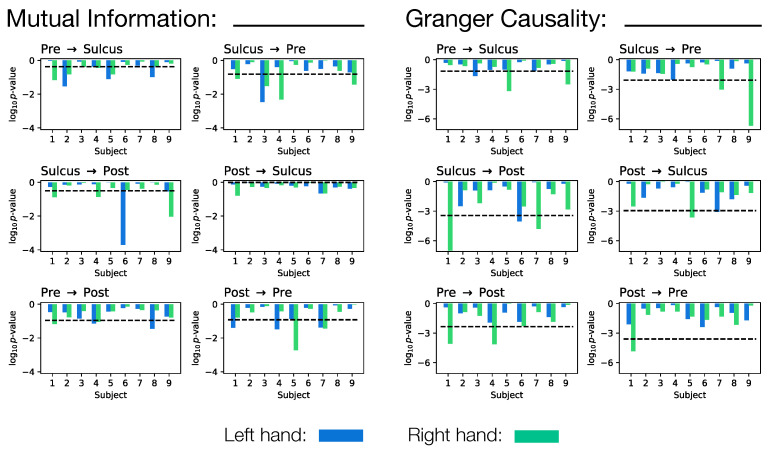
Individual and aggregated *p*-values obtained using the MI (**left panels**) and GC (**right panels**) tests. Each panel reports the results for a pair of regions (see titles). Inside each panel, the blue and green bars indicate the *p*-values obtained for each participant, respectively, for the left and right hand. Finally, the horizontal dashed lines report the aggregated *p*-value, as described in Section 2.3.

Table 3 reports the aggregated $\log_{10}$ of the *p*-values obtained by the MI (top half) and GC (bottom half) over the complete time series, i.e., akin to the top part of Table 1. As in the previous case, individual results for all participants and sides can be found in Figure A7. Note that, as opposed to the previous case in which results were reported as Z-Scores, here a value smaller than −2 (or −3) indicates a statistically significant result, for a significance level of α=0.01 (or, respectively, α=0.001).

It can be appreciated that the MI completely fails at detecting any statistically significant relationship, with all *p*-values larger than 0.1 (i.e., $\log_{10}$*p*-values >−1). On the other hand, the GC retrieves results very similar to the IIG causality on the tapping-only time series, albeit with a low statistical significance. Notably, the GC does not detect any propagation of information from the precentral to the sulcus regions, even though this was the second strongest link according to the IIG.

**Figure A8:**
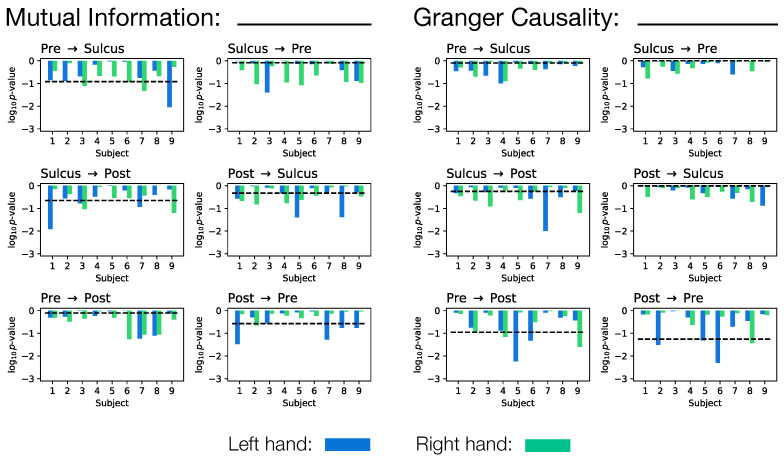
Individual and aggregated *p*-values obtained using the MI (**left panels**) and GC (**right panels**) test on the randomly shuffled data. Meaning of panels, bars, and horizontal lines as in Figure A7.

For the sake of completeness and in order to exclude false positives, Figure A8 further reports the same results when the original time series are randomly shuffled. The results are in line with what expected, with aggregated $\log_{10}$*p*-values never smaller than −1. At the same time, it can be noted how results for some individual participants reach statistically significant values.

### 3.3. Temporal Evolution

As a final point, we here evaluate the temporal evolution of the relationships detected by the IIG causality. Note that this is a challenging task due to the limited quantity of information available, which further precludes the use of MI and GC in this specific case.

**Figure A9:**
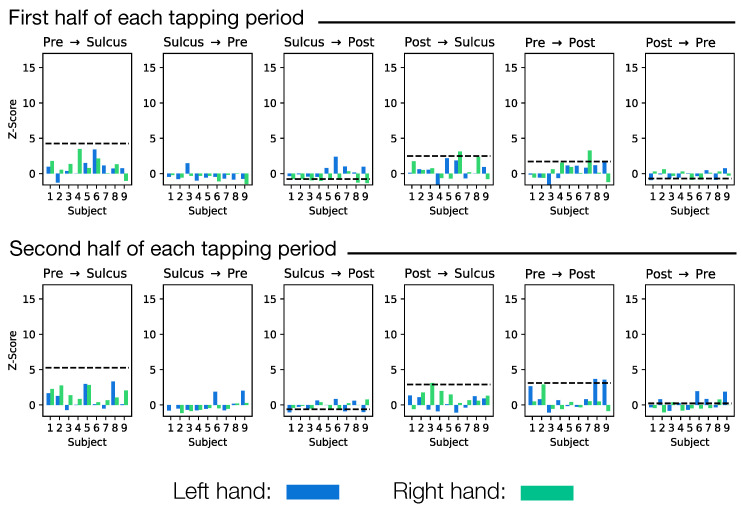
Individual and aggregated Z-Scores obtained using the IIG causality test. Each panel reports the results for a pair of regions (see titles). Top and bottom panels, respectively, correspond to considering the activity in the first and second halves of each tapping period, in the contralateral hemisphere. Inside each panel, the blue and green bars indicate the Z-Score obtained for each participant, respectively, for the left and right hand. Finally, the horizontal dashed lines report the aggregated Z-Score, as described in Section 2.3.

We initially evaluated whether the functional connectivity was different in the first 15 s of each tapping period, considering the activity of the contralateral hemisphere, compared to the last 15 s of it. The results, presented in Figure A9, are qualitatively the same; in other words, flows of informations do not significantly change within each tapping task.

**Figure 4:**
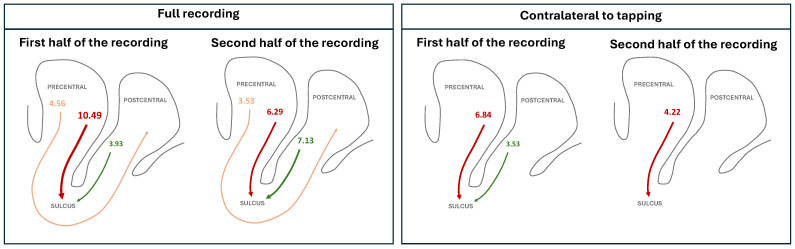
Graphical representations of the main flows of information detected by the IIG causality. The left box compares the first (left graph) and second halves (right graph) of each recording session; conversely, the right box presents the same comparison, but considering only the activity contralateral to the tapping. See Table 4 and Table 5 for full numerical values. Line thickness is proportional to the aggregated Z-Score, also reported numerically.

**Figure A10:**
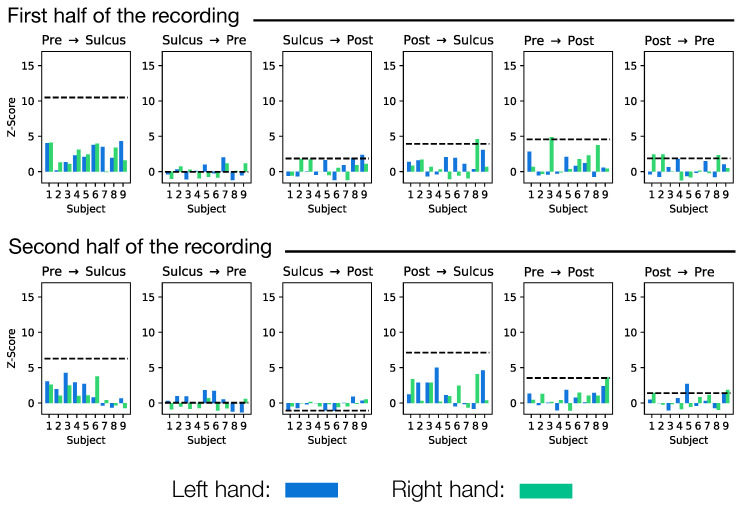
Individual and aggregated Z-Scores obtained using the IIG causality test. Each panel reports the results for a pair of regions (see titles). Top and bottom panels, respectively, correspond to considering the activity in the first and second halves of each recording session. Inside each panel, the blue and green bars indicate the Z-Score obtained for each participant, respectively, for the left and right hand. Finally, the horizontal dashed lines report the aggregated Z-Score, as described in Section 2.3.

We next move to the analysis of the whole time series by firstly considering the first vs. the second half of it—note that here we do not distinguish between periods of tapping and resting, and thus evaluate the average of them. The results are synthesised in Table 4 (with a graphical representation in the left box of Figure 4), and further extended in Figure A10. Notably, while the flow precentral → sulcus is dampened, the opposite occurs in the case of postcentral → sulcus, with the aggregated Z-Score increasing from 3.93 to 7.13.

**Figure A11:**
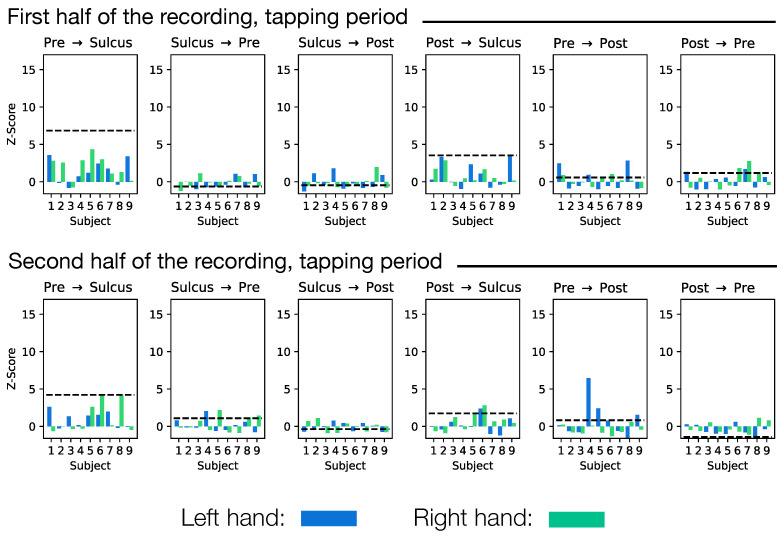
Individual and aggregated Z-Scores obtained using the IIG causality test. Each panel reports the results for a pair of regions (see titles). Top and bottom panels, respectively, correspond to considering the activity in the first and second halves of each recording session, considering only the activity contralateral to the tapping. Inside each panel, the blue and green bars indicate the Z-Score obtained for each participant, respectively, for the left and right hand. Finally, the horizontal dashed lines report the aggregated Z-Score, as described in Section 2.3.

We finally analyse the first half of the activity in the hemisphere contralateral to the tapping task, vs. the second half of it—this is, thus, equivalent to only considering the activity directly associated with the tapping, and how it evolved from the beginning to the end of the recording session. As before, the results are synthesised in Table 5 (with the corresponding graphical representation in the right box of Figure 4), and extended in Figure A11. While the same drop of information flow, i.e., of Z-Score, can be observed in the precentral → sulcus case, the postcentral → sulcus one is also reduced (from a Z-Score of 3.53 to 1.74).

In synthesis, throughout the recording session, the connection from the postcentral gyrus to the sulcus is strengthened, but only during the periods of time in which the hemisphere is not directly involved in the tapping; the opposite happens during the tapping. Following the intuition sketched in Section 3.1, this suggests that the postcentral gyrus progressively shares more information to the sulcus throughout the duration of the recording session, but also that the latter is more and more bypassed, in favor of a direct and fast connection postcentral → precentral, when the tapping is executed. In other words, while the expectation of performing the tapping results in more information transmitted to the sulcus, the neural circuits learn to use a more direct route postcentral → precentral when the task is actually carried out.

## 4. Discussion and Conclusions

This paper is to our knowledge the first attempt to elucidate the propagation of functional information between M1 of the precentral gyrus; Area 3a of the fundus of the central sulcus; and SI Areas 3b, 1, and 2 of the postcentral gyrus, which should sustain the primary substrate for planning, learning, and plasticity of tapping movements. We approached this question using the Information Imbalance Gain (IIG) causality test, which assesses the information present in the activity of fMRI voxel series in one region, in terms of their capacity for predicting the future activity in a second one. Overall IIG found an intense propagation of information from the precentral and postcentral regions towards the sulcus within the corresponding hemispheres, both during the tapping task of the contralateral hand and during periods in which that hand was at rest but the ipsilateral hand was tapping. This pattern applied for both hands, although it was less pronounced for the non dominant one. Additionally, a weaker but statistically significant reciprocal flow of information was detected between the precentral and postcentral regions. In contrast, the Mutual Information algorithm completely failed to identify any statistically significant relationship between precentral, postcentral, and sulcus regions. Granger Causality produced results broadly similar to those of IIG for the tapping-only time series, although with a low statistical significance. This discrepancy is likely due to the limited number of time series used here, which, as discussed before, constrains the performance of MI and GC algorithms.

It is not to be assumed that the relationships found here between areas rely on direct cortico-cortical connections. The last track tracing study existing in monkey [13] defined non-existing cortical connections between Area 3a in the sulcus towards or from Area 4 in the precentral region, and neither between Areas 3a and 3b in the postcentral region. However, it defined a strong connection between Areas 4 and 2 (and not between Areas 4 and 3b and 1), and between Areas 1 and 2 with 3a. Studies of effective connectivity in resting state [52] found strong effective values of functional connectivity between all areas of M1 and SI, specially between Areas 3a and 3b towards Areas 1 and 2. Interestingly, they did not find strong connectivity from area 4 to Area 3a either. What we have described contradicts both studies, in that we find propagation from precentral Area 4 to Area 3a in the fundus of the sulcus, but supports them in the propagation from other SI areas towards Area 3a. In the first set of experiments of our study, we included in the voxel series of the sulcus a small portion of underlying white matter-activated voxels just underneath the fundus gray matter. In that white matter region, the sulcus could be conveying arched U connecting fibers from the postcentral gyrus to Area 4 and vice versa, which could lead to BOLD fMRI activity interpreted as activity towards the sulcus. In a second set of experiments, we discarded those white matter fMRI voxels and included in the IIG algorithm only those activated voxels located in the grey matter of the sulcus. This has produced quite similar results in terms of the flow of information from precentral regions towards the sulcus, and significant but weaker propagation between postcentral regions and the sulcus. Our interpretation is that the precentral and postcentral regions are directing information towards the sulcus Area 3a and also between them, both during the ipsilateral and contralateral tasks. In general, IIG causality results must not directly be interpreted as a proof for structural connectivity. On the one hand, the IIG metric (and more generally, any functional metric) describes information flows, which can happen directly (i.e., supported by fibers), or indirectly across other regions. On the other hand, differentiating between these two alternatives is a challenging task due to the fact that real neuronal transmission between areas takes place much faster than the resolution of the fMRI recordings. As discussed in Section 3.1 and Appendix D, we cannot define whether the propagation of activity between pre- and postcentral regions is performed with direct connections or via sulcus, because of the temporal resolution of the time series. However, we can define that these pre- and postcentral gyri are exchanging information during the tapping tasks, and that the activity in the precentral and postcentral regions seems to be mediated by the sulcus, given their strong causality connection with this region. This raises the question of the role of Area 3a in the transmission during the task, since the causality connection in the other direction, i.e, sulcus towards pre- and postcentral gyri, is either very weak, or, as discussed previously, is so fast that is not seen by the algorithm.

Another interesting finding is the persistence of this flow of activity between the pre- and postcentral regions and the sulcus while the hemisphere is not directly involved in the task, but the task is being performed by the ipsilateral hand. This is coherent with the meta-analysis by Witt et al. [73], which generated Activation Likelihood Estimate maps of the main effects of all finger tapping task variations, and found robust concordance in bilateral sensorimotor cortices while performing a right handed multifinger tapping task. A second explanation could rely on the imagery of the next expected movement of the resting hand. In relation to this, recent fMRI results [39] have evidenced that the planning of the finger movement activates zones in Area 4 and in SI with peaks of activity that correlate those that are produced during real training. Also, Chen et al. [63] have reported during motor imagery forward and backward effective Granger Causality connectivity between the supplementary motor area and the contralateral primary and secondary somatosensory cortex (SI), and the primary motor cortex (M1). Attentional effects have also been demonstrated for SI. Braun et al. demonstrated that the hand representation within the SI is not statically fixed but is dynamically modulated by top-down mechanisms to support task requirements, which concedes a greater capacity for modulation of the functional cortical organization [91].

It is further worth highlighting the changes that occur during the whole time of task execution in terms of the flow of activation between regions. As described in Section 3.3, the connection from the postcentral gyrus to the sulcus is strengthened during the recording session, but only during the periods of time in which the hemisphere is not directly involved in the tapping; the opposite happens during the tapping. This suggests that the postcentral gyrus progressively shares more information to the sulcus throughout the duration of the recording session, but also that the latter is more and more bypassed, in favor of a direct and fast connection postcentral → precentral, when the tapping is executed. In other words, while the expectation and planning of performing the tapping results in more information transmitted to the sulcus, the neural circuits learn to use a more direct route postcentral → precentral when the task is actually carried out.

These observations reinforce the idea that enhanced training on motor tasks has an effect on the level of activation observed in the primary sensorimotor cortex raised by functional neuroimaging studies [92,93,94,95]. Those works have demonstrated an initial decrease in primary sensorimotor cortical activation contralateral to the moving hand during motor skill acquisition, followed by an enlargement in activation in this same region during the course of motor training, which has been shown to be sustained for up to four weeks post-training. Motor skill acquisition has been suggested to occur in two discrete stages: the first being a fast learning, initial, within-session improvement phase, and the second being a slow learning phase, consisting of delayed, incremental gains in performance during continued practice [93]. Those observations are based on measurements of quantitative activity. Our results go further and demonstrate changes in the direction of flow of information that are happening during the fast, within-session first stage of movement learning. This is consistent with the rising idea that the primary sensorimotor cortex is not only an executive locus for simple voluntary movements [96], but instead participates in the processing of complex sequential tapping tasks [33,36,97,98,99,100,101] and in the processing of bimanual movements [102].

In macaque, Area 3a contains a complete representation of deep receptors of the contralateral body, with a topographic organization less precise as that of area 3b. Most individual body parts are represented in more than one cortical territory. The receptive fields for neurons are large, and the forelimb, hand, and digit representations have a large cortical magnification factor [103]. Studies on cortical connections of electrophysiologically defined locations in Area 3a were described in the marmoset monkey [104], reporting that Area 3a has much denser connections with motor and posterior parietal areas of the neocortex than with somatosensory areas. The forelimb representation in Area 3a has very broad, topographically mismatched connections with the forelimb representation in other fields and other body part representations (such as the face representation), while the foot representation has topographically matched connections mainly with the foot representation in other cortical areas. This suggested that, at least in primates, Area 3a appears to be involved in integrating somatic and vestibular inputs with the motor system, maintaining posture and forelimb position, and regulating velocity of limb movement. Finally, studies of plasticity in motor and somatosensory cortex of adult mammals using a variety of different manipulations [105,106,107,108,109,110,111] demonstrate that the cortical zone of reorganization was greater in Area 3a than in Area 3b in the same animal, thus reflecting use-dependent processes for that individual. We cannot infer such representational changes in Area 3a of humans during learning, but the steady and increased flow of activity towards the region of the sulcus might be reflecting a supporting role for that kind of integration processes between M1 and SI. This role could be related to the fact that learning the movement requires the amplification of the somatotopic representations, including that necessary to combine movement of several fingers, which is a property that is present in Areas 4 and 3a. It is possible that Area 3a is then contributing to the maintenance of representational plasticity, as a sort of connection hub. Our results additionally indicate that, during the tapping task, rostral regions to the precentral sulcus are activated—see Figure A1. According to Ruland et al. [112], these regions likely correspond to areas 6d1, 6d2, and 6v3 of the premotor cortex, which have been functionally associated with arm and hand movements, and object and temporal prediction. Considering their associative and structural connectivity, it remains plausible that these areas constitute a higher-level hub relative to the somatomotor network described here. Although the analysis of functional connectivity between these premotor regions and M1 and SI was beyond the scope of the present study, it represents an interesting direction for future research.

As a final point, it is important to highlight some limitations of the present study: First of all, the sample size is limited, and a larger cohort would be desirable to minimize potential biases [113,114]. This limitation was partly compensated by the use of a Z-Score aggregation procedure, which enhances robustness by combining individual subject-level effects into a standardised metric, reducing variability and increasing sensitivity to true activations across participants. Still, future studies incorporating a larger sample would further strengthen the generalizability of our findings. Secondly, the age of participants ranged from 25 to 60 years, with eight individuals younger than 47 and only one aged 60. This heterogeneity might have introduced some variability related to age-dependent plasticity or cerebral vascularization. We, nevertheless, made a special effort to recruit volunteers with highly homogeneous cognitive and sociocultural profiles, as all the participants were medical doctors working at the same hospital. This homogeneity helps minimising confounding factors related to education, professional expertise, and cultural background, therefore reducing potential biases in task performance and neural activation patterns.

## Figures and Tables

**Table 1:** Aggregated Z-Scores of the IIG causal relations between the precentral gyrus, the sulcus, and the postcentral gyrus. The top, middle, and bottom parts, respectively, correspond to the analysis of the whole time series, to the analysis of the activity of the hemisphere contralateral to the tapping, and of that ipsilateral to it. See Figure A3 for individual values.

Full recording:				
		To:		
		Precentral	Sulcus	Postcentral
**From:**	**Precentral**	-	13.97	6.17
	**Sulcus**	0.27	-	0.17
	**Postcentral**	3.02	10.41	-
**Contralateral to tapping:**				
		**To:**		
		**Precentral**	**Sulcus**	**Postcentral**
**From:**	**Precentral**	-	10.45	1.62
	**Sulcus**	−0.37	-	−0.58
	**Postcentral**	1.36	8.01	-
**Ipsilateral to tapping:**				
		**To:**		
		**Precentral**	**Sulcus**	**Postcentral**
**From:**	**Precentral**	-	5.92	2.51
	**Sulcus**	0.71	-	0.68
	**Postcentral**	0.37	4.50	-

**Table 2:** Aggregated Z-Scores of the causal relations to and from the sulcus, as obtained through the IIG causality. Values inside the parentheses indicate the Z-Scores obtained when voxels corresponding to white matter are not included in the activity of the sulcus—thus only considering voxels belonging to the cortex. See Figure A6 for individual values.

Direction	Full Rec.	Contralateral	Ispilateral
From **sulcus** to **pre**:	−1.77 (0.27)	0.08 (−0.37)	−1.29 (0.71)
From **sulcus** to **post**:	−2.17 (0.17)	−2.35 (−0.58)	−0.82 (0.68)
From **pre** to **sulcus**:	11.99 (13.97)	7.27 (10.45)	6.76 (5.92)
From **post** to **sulcus**:	10.00 (10.41)	3.81 (8.01)	5.52 (4.50)

**Table 3:** Aggregated $\log_{10}$ of the *p*-values between precentral, sulcus and postcentral regions, as yielded by the MI (upper table) and the GC (bottom table). See Figure A7 for individual values.

Mutual Information:				
		To:		
		Precentral	Sulcus	Postcentral
**From:**	**Precentral**	-	−0.37	−0.96
	**Sulcus**	−0.81	-	−0.50
	**Postcentral**	−0.92	−0.01	-
**Granger Causality:**				
		**To:**		
		**Precentral**	**Sulcus**	**Postcentral**
**From:**	**Precentral**	-	−1.16	−2.35
	**Sulcus**	−2.08	-	−3.44
	**Postcentral**	−3.60	−2.94	-

**Table 4:** Evolution of the Z-Scores yielded by the IIG test when comparing the first and second halves of each recording session (respectively, before and after the arrows). See Figure A10 for individual values.

		To:		
		Precentral	Sulcus	Postcentral
**From:**	**Precentral**	-	10.49 → 6.29	4.56 → 3.53
	**Sulcus**	−0.02 → 0.03	-	1.86 → −1.07
	**Postcentral**	1.88 → 1.39	3.93 → 7.13	-

**Table 5:** Evolution of the Z-Scores yielded by the IIG test, comparing the first and second halves of each recording session, and considering only the periods of activity contralateral to the tapping. See Figure A11 for individual values.

		To:		
		Precentral	Sulcus	Postcentral
**From:**	**Precentral**	-	6.84 → 4.22	0.57 → 0.82
	**Sulcus**	−0.63 → 1.08	-	−0.46 → −0.37
	**Postcentral**	1.17 → −1.40	3.53 → 1.74	-

**Figure A2:**
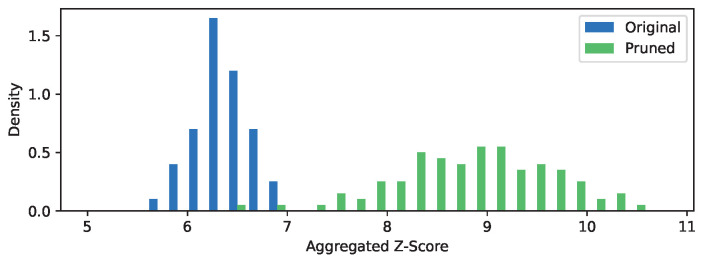
Histograms of the Z-Scores obtained using the IIG test, for the relationship between pre- and postcentral gyri, using the complete recordings—see top of Table 1. Blue bars correspond to the use of all voxels of the latter region and green bars to a random pruning of these voxels to achieve the same cardinality as the sulcus.

**Figure A5:**
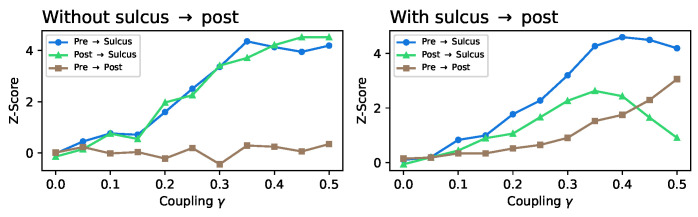
Evolution of the intensity of the causal connection detected by the IIG causality, when analysing time series generated with the models of Equations (A1) (**left panel**) and (A2) (**right panel**). The causality, measured through the average Z-Score (see Section 2.3), is reported here as a function of the coupling strength γ.

**Figure A6:**
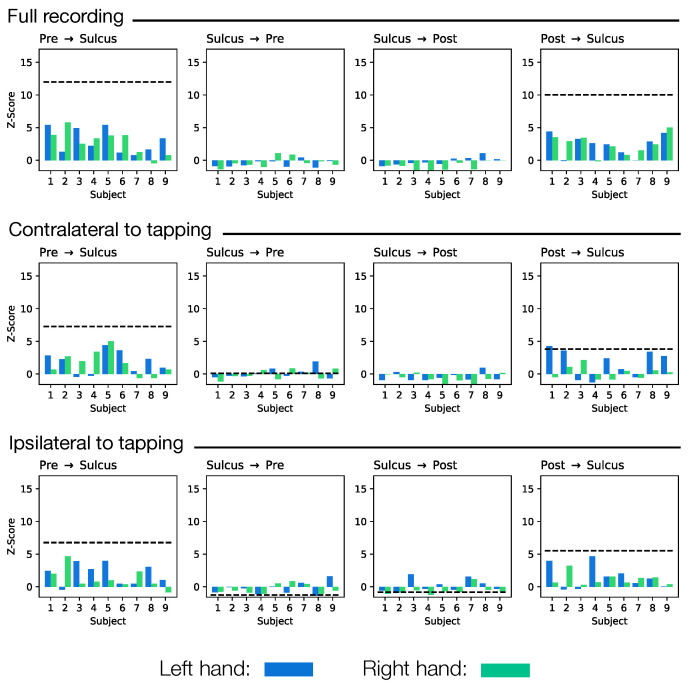
Individual and aggregated Z-Scores obtained using the IIG causality test, for relations to and from the sulcus when only cortex voxels are included in its definition. Each panel reports the results for a pair of regions (see titles). From top to bottom, each set of panels, respectively, corresponds to the whole recording; to the activity of the hemisphere contralateral to the tapping; and to the activity ipsilateral to the tapping. Inside each panel, the blue and green bars indicate the Z-Score obtained for each participant, respectively, for the left and right hand. Finally, the horizontal dashed lines report the aggregated Z-Score, as described in Section 2.3.

## Data Availability

The raw data supporting the conclusions of this article will be made available by the authors upon reasonable request.

## Abbreviations

The following abbreviations are used in this manuscript:

aMRI	anatomical Magnetic Resonance Imaging
fMRI	functional Magnetic Resonance Imaging
GC	Granger Causality
IIG	Information Imbalance Gain
M1	Motor Cortex
MI	Mutual Information
SI	Somatosensory Cortex

## Appendix A. Subject Characteristics

**Table A1:** Subject demographics and characteristics.

Subject	Sex	Age (Years)	Handedness
1	Female	35	Right handed
2	Male	60	Right handed
3	Male	47	Right handed
4	Female	25	Right handed
5	Male	33	Right handed
6	Male	41	Right handed
7	Female	36	Right handed
8	Female	29	Right handed
9	Male	28	Right handed

**Table A2:** Number of voxels composing each region considered in this study for the nine available subjects. Numbers in parenthesis report the percentage of those voxels that showed a statistically significant activity during the tapping task, as yielded by a *t*-test with a significance level of 0.05.

Subject	Left Hemisphere		Right Hemisphere	
	Precentral	Postcentral	Precentral	Postcentral
1	15,362 (35%)	13,295 (31%)	13,340 (33%)	15,318 (40%)
2	19,521 (19%)	17,769 (39%)	19,969 (25%)	20,105 (30%)
3	14,793 (42%)	10,074 (35%)	16,466 (33%)	8778 (23%)
4	19,464 (24%)	15,265 (30%)	18,680 (19%)	15,007 (26%)
5	22,418 (16%)	17,669 (38%)	21,306 (22%)	15,399 (32%)
6	26,241 (18%)	23,027 (11%)	23,117 (28%)	17,880 (26%)
7	20,210 (34%)	13,321 (36%)	20,668 (28%)	12,742 (30%)
8	23,961 (14%)	15,266 (27%)	20,031 (29%)	15,285 (23%)
9	23,541 (23%)	15,226 (29%)	17,694 (32%)	15,481 (27%)
	**Sulcus**	**Sulcus (Cortex)**	**Sulcus**	**Sulcus (Cortex)**
1	1206 (13%)	601 (38%)	2147 (30%)	948 (32%)
2	5409 (8%)	1809 (26%)	5424 (9%)	1675 (17%)
3	3418 (24%)	1338 (58%)	4076 (23%)	1274 (16%)
4	4424 (1%)	1605 (12%)	4531 (17%)	1967 (24%)
5	4844 (1%)	1735 (11%)	5070 (16%)	1584 (20%)
6	8084 (5%)	2703 (17%)	8806 (11%)	2626 (15%)
7	6903 (11%)	2091 (34%)	6170 (24%)	1838 (42%)
8	7226 (16%)	2258 (26%)	7208 (18%)	1964 (21%)
9	5728 (5%)	2230 (24%)	7614 (34%)	2089 (30%)

## Appendix B. Statistical Variability of IIG and Its Dependence on the Problem Size

As discussed in Section 2.2, calculating the Z-Score for the IIG test requires the comparison of the observed Information Imbalance, with what obtained when randomly shuffling the data for the causing region. This introduces stochasticity in the calculation, i.e., different Z-Scores can be obtained when analysing the same data set.

This is illustrated in Figure A2. Specifically, the blue bars report the histogram obtained by performing the calculation of the Z-Score between the pre- and postcentral gyri using the full recordings. While the value reported in Table 1 for this case is 6.17, Figure A2 illustrates how such value has actually to be understood as a probability distribution. Importantly, the variability of the obtained Z-Scores is generally low—in the case at hand, the standard deviation is of 0.257.

Another relevant characteristic of the IIG test is that it allows to compare regions with a highly different number of voxels—see Table A2. In order to understand how such number affects the detection of the causality, the green bars of Figure A2 report the histogram of the Z-Scores obtained when a random subset of voxels of the postcentral gyrus are used in the calculation, with a cardinality equal to that of the sulcus. In other words, we randomly prune the postcentral region to simulate it having the same number of voxels as the sulcus—i.e., as the smallest region here considered. The obtained Z-Score is not hindered by such pruning, but instead slightly increases. This nevertheless comes at the cost of a higher variability (standard deviation of 0.774), as now the selection of the voxels is also randomised. From the viewpoint of this study, this result confirms that the heterogeneity in size between regions is not a problem when the causality is calculated using the IIG test.

## Appendix D. Synthetic Model of Confounding Effects

In order to illustrate how confounding effects can explain the results illustrated in Table 1, we here resort to a synthetic model comprising three regions, akin to the sulcus, precentral, and postcentral ones. Each one of them is represented by 100 time series, representing 100 voxels, respectively, denoted by $s_{i}$, $pre_{i}$, and $post_{i}$. These are initialised using a synthetic model of fMRI dynamics, encompassing three steps: (i) the generation of raw time series using a VAR model and additive Gaussian noise; (ii) their convolution through the canonical hemodynamic response function (HRF), describing the blood and nutrient requirements of neurons as a function of their activity, defined as a mixture of two gamma functions; and finally, (iii) the downsampling of the time series, to better reflect the technical limitations of fMRI machines. The full process for generating these synthetic time series is described in Refs. [115,116].

Next, these time series are linearly coupled according to the following rule:

(A1) $$s_{i}\left(t+1\right)=\left(1-2\gamma \right)s_{i}\left(t+1\right)+\gamma pre_{i}\left(t\right)+\gamma post_{i}\left(t\right).$$

The dynamics of each voxel of the sulcus is then the result of adding what observed in the precentral and postcentral, one step back in time, and with an intensity equal to γ (here acting as a coupling intensity). As is to be expected, the IIG metric detects two causality relations, i.e., precentral → sulcus and postcentral → sulcus, with an intensity proportional to γ—see left panel of Figure A5. Also as expected, no causal connection can exist between the precentral and postcentral regions (see brown line), as these are not connected in Equation (A1).

Next, we modify the model to include an additional coupling:

(A2) $$\begin{array}{c} s_{i}\left(t+1\right)=\left(1-2\gamma \right)s_{i}\left(t+1\right)+\gamma pre_{i}\left(t\right)+\gamma post_{i}\left(t\right), \end{array}$$

(A3) $$\begin{array}{c} post_{i}\left(t\right)=\left(1-\gamma \right)post_{i}\left(t\right)+\gamma s_{i}\left(t\right). \end{array}$$

Note that, here, the postcentral region receives information from the sulcus without any delay, i.e., the dynamics of the former at time *t* is a function of the dynamics of the latter also at time *t*. On the other hand, the sulcus receives information from the precentral region with a delay of one time step. The result is the detection of a spurious causality relation precentral → postcentral, which is the result of the intermediation of the sulcus—see right panel of Figure A5, brown line. As a consequence, a causal relation precentral → postcentral may be due either to a direct or a sulcus-mediated information flow, the two alternatives being impossible to be discriminated without further data.
